# Upstream, downstream: *n*emo learns where plant regulatory sequences act

**DOI:** 10.1093/plphys/kiag629

**Published:** 2026-08-24

**Authors:** Neeta Lohani, William J W Thomas

**Affiliations:** Assistant Features Editor, Plant Physiology, American Society of Plant Biologists, Rockville, MD 20855-2768, United States; Department of Biotechnology, Thapar Institute of Engineering and Technology, Patiala, Punjab 147004, India; Assistant Features Editor, Plant Physiology, American Society of Plant Biologists, Rockville, MD 20855-2768, United States; School of Biological Sciences, The University of Western Australia, Perth, Western Australia, Australia

A plant's genome not only contains gene sequences which encode proteins and RNAs, but also the instructions of when and where to make them. This regulatory grammar is complex, and researchers still cannot easily decipher it. Transcription factors (TFs) are proteins that bind to specific sequences within cis-regulatory elements and recruit the core machinery that starts transcription. TFs are responsible for writing much of the regulatory grammar that controls gene expression in plants. Genetic variations at TF binding sites also account for a large portion of heritable trait variations in crops such as maize (Engelhorn et al. 2025). The position and genomic context of a TF binding site can be just as important as the binding site sequence itself. Transcription regulation in plants is unusually local, with around 75% of accessible chromatin regions sitting immediately outside the transcribed regions, most of them within 2 kb upstream of the transcription start site (TSS) or 1 kb downstream of the transcription termination site (TTS) (Maher et al. 2018). Voichek et al. (2024) showed that the C2C2-GATA family of TFs strongly upregulate genes but only when their binding sites are within 500 bp downstream of the TSS. Many other plant TF families also have preferred binding positions relative to the TSS. But preferring a position is not the same as requiring it, and for most TF families we still do not know whether their functions actually depend on where they bind. Part of the difficulty is that regulatory elements often cluster together into modules, so a preferred position can simply be a byproduct of the company a motif keeps (Acevedo-Luna et al. 2016).

The emergence of deep learning offers a promising new route to identify the complex regulatory grammar within plant genomes. For example, convolutional neural networks (CNNs) can learn informative DNA motifs without being told what to look for. Researchers can then probe a trained CNN model to recover the logic it captured and the sequences it learned. However, polyploid crops complicate this because their complex, duplicated, and highly redundant genome blurs read mapping and creates arbitrary similarities between related genes. Those similarities inflate performance estimates and force the model to memorize evolutionary artifacts rather than learn the desired biology (Washburn et al. 2019).

Recently in *Plant Physiology*, Rockenbach et al. (2026) describe *n*emo (***n****apus*  **e**xpression **mo**del), a branched CNN sequence-to-expression model that predicts median gene expression from promoter and terminator sequences in allotetraploid oilseed rape (*Brassica napus*) and the model plant Arabidopsis (*Arabidopsis thaliana*). Rather than adapting an existing model, the authors specifically designed *n*emo to handle polyploid genomes. They did so by generating expression values for evolutionarily related gene copies (homoeologs) and used these values to discard copies with identical sequences. They also masked gene coding regions and partitioned genes into distinct folds so the model could not exploit similarity between training and test data. Their state-of-the-art model explained 53 to 54% of the variation in median expression across unseen gene families and outperformed four existing sequence-to-expression model architectures which they implemented on the same data, including Xpresso (Agarwal and Shendure 2020) and PhytoExpr (Li et al. 2024). One key design choice for *n*emo was thought to explain its performance. Where most models use non-overlapping max-pooling, *n*emo uses average pooling over overlapping windows. This feature preserves finder spatial detail, and it signals that the exact position of a sequence matters for accurate prediction.

Training the model on a polyploid genome made *n*emo more robust, contrary to the expectation that complex, redundant genomes hurt model performance. For example, when *n*emo was trained on polyploid *B. napus*, it kept its accuracy when tested on Arabidopsis. However, when *n*emo was trained on diploid Arabidopsis, it lost accuracy when tested on *B. napus*. Part of this asymmetry reflects data quality, since the Arabidopsis genome is extremely well-characterized and has a highly curated annotation. This allowed the model trained on Arabidopsis to achieve higher overall performance when applied to its own genome. However, this accuracy did not transfer over to *B. napus*. Instead, the *n*emo model trained on polyploid data was able to learn from the many near-identical sequences present in the *B. napus* genome. This taught the model how small sequence changes shift expression and provided the robustness that *n*emo boasts.

To ground truth *n*emo, the authors compared gene expression predicted by the model to experimentally measured expression values from RNA-Seq data. They first compared two *B. napus* varieties, Express617 and ZS11, which represent different ecotypes, and selected 100 genes with extreme differences in predicted expression. *n*emo was able to accurately identify which variety had higher expression for 77.5% of cases. These differentially expressed genes were also enriched for nearby structural variants, highlighting the possible role of structural variation in explaining expression differences. However, genome-wide mapping will be required to confirm this link.

The authors then interrogated what *n*emo had actually learned. They scored each sequence position with Shapley values, a measure of how important each position is to the model's prediction, revealing sequences that contributed most to *n*emo's prediction. They then grouped genes by the TF families known to bind their promoters, drawing on DNA affinity purification sequencing (DAP-seq) data (O’Malley et al. 2016). For several families the importance profiles matched the experimental binding preferences in both species. Most convincingly, *n*emo recovered the hallmark C2C2-GATA signal. Inserting C2C2-GATA motifs in silico raised predicted expression, but only if inserted close to the TSS. This reproduced the position-specific effect that Voichek et al. (2024) demonstrated experimentally. The model also assigned TCP factors a strong positive effect when near the TSS but predicted the opposite effect for bHLH and bZIP factors, depending on whether their motifs landed upstream or downstream of the TSS.

One major question looming over *n*emo was if the model was responding to features within motifs, such as GC content, rather than the specific motif itself. To test this, the authors placed three decoys at the same position as the real motif: a scrambled version, a reversed version and a random sequence. When comparing the effects, true motifs were found to shift expression more so than decoys. Although for AP2/EREBP factors, scrambled decoys influenced expression almost as much as the intact motifs. These motifs are known to be GC rich and it was proposed that the model had responded to their GC composition rather than their exact sequence. Openly acknowledging this weakness made the cleaner results, such as those for C2C2-GATA and TCP families, more convincing.

The model's apparent shortcomings proved just as useful as its successes. *n*emo consistently underpredicted the expression of genes near super-enhancers and overpredicted expression of genes carrying repressive H3K27me3 marks (Fig. 1). Both “errors” have the same root cause, because the model reads only proximal sequences and cannot see distal enhancers or epigenetic silencing. This blind spot actually flags genes that may sit under more complex regulatory control. As a result, what was first thought to be an error of the model, became an informative discovery tool which can distinguish genes governed by local regulatory sequences from those that answer to distal or epigenetic regulation.

**Figure 1:**
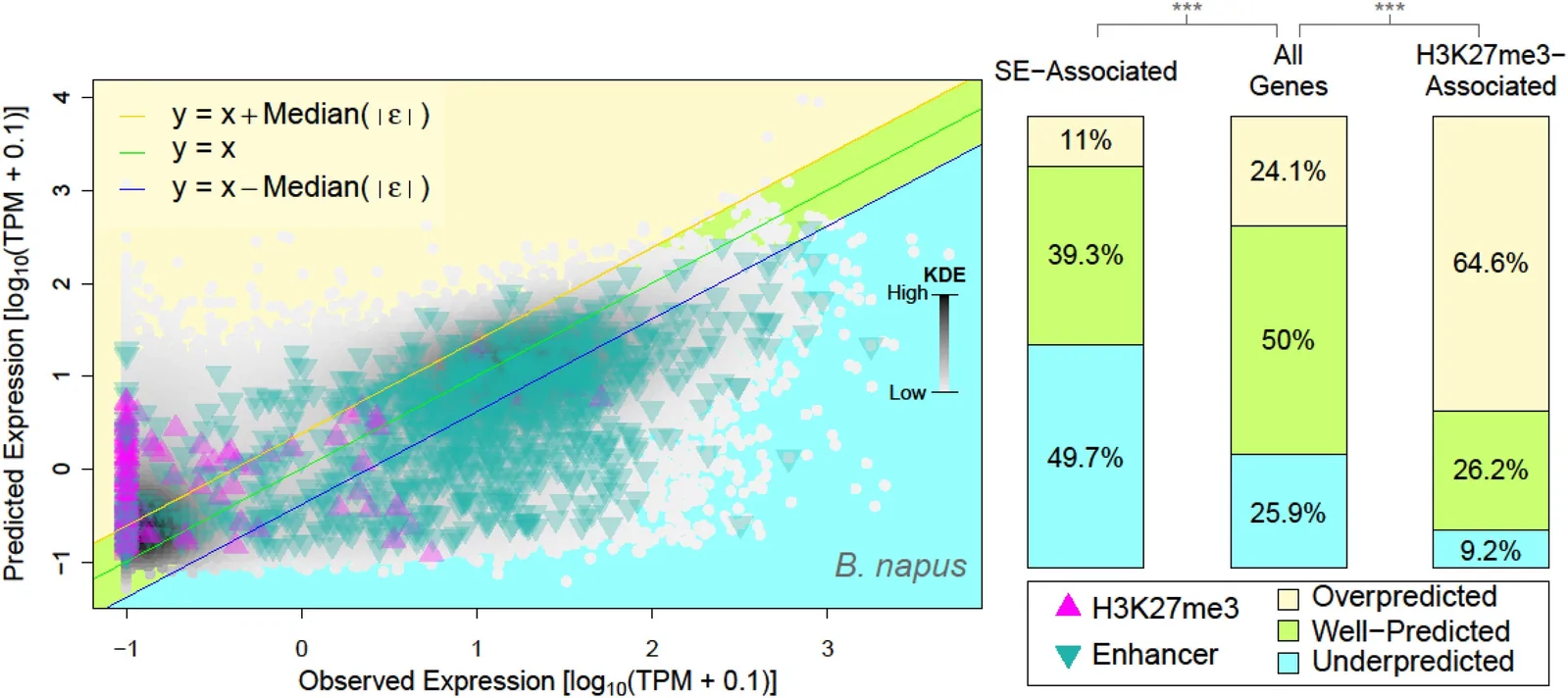
Prediction errors reveal genes under complex regulatory control. Predicted versus observed median gene expression in *B. napus*. Green line: perfect prediction (y = x); gold and blue lines: well-predicted zone boundaries (y = x ± median absolute error). Pink triangles: H3K27me3-associated genes; teal triangles: super-enhancer-associated genes. Stacked bars show that H3K27me3-associated genes are enriched among overpredicted genes, while super-enhancer-associated genes are enriched among underpredicted genes (χ^2^ tests, both *P* < 2.2 × 10^−16^). Adapted from Rockenbach et al. (2026).

Together these results show that a carefully built CNN can learn not only which motifs matter but where they must sit to act, even in a complex polyploid crop. The model's limitations point to where the field may be progressing. *n*emo predicts median expression from proximal sequences, so the tissue-specific, distal, and epigenetic control behind many important crop traits still lies beyond its view. The integration of chromatin or histone-mark data is a clear path to improving the model's performance. Its sharpest predictions double as experiments in waiting, since the opposing effects of bHLH and bZIP motifs across the TSS could be tested directly in synthetic promoters. As breeders move toward precise editing of regulatory sequences, models like *n*emo could help predict which changes will fine-tune expression in the desired direction, bringing the targeted engineering of gene regulation in crops within closer reach.

## Recent related articles in Plant Physiology

Zhu et al. (2025) built a machine-learning tool that predicts transcription factor–target gene interactions in soybean, extending sequence-based modeling of regulatory control to a major legume crop.

Zenker et al. (2025) analyzed binding data for hundreds of Arabidopsis transcription factors and identified a constrained set of conserved binding motifs shared across families, defining the experimental grammar that models such as *n*emo aim to recover from sequence alone.

## Data Availability

No new data were generated or analyzed in support of this article.
